# Society Meetings

**Published:** 1903-12

**Authors:** 


### SOCIETY MEETINGS.

The Medical Society of the County of Chautauqua, will hold its semiannual meeting at Fredonia, December 9, 1903, under the presidency of Dr. W. J. French, Hamlet. Papers are promised by Drs. John Parmenter, Buffalo; A. D. Lake, Gowanda; Ellis H. Storms, Cherry Creek; C. H. Richards and W. H. Vosburg, Dunkirk. The profession of adjoining counties is invited to participate in the proceedings.

---

The Medical Society of the State of New York will hold its ninety-eighth annual meeting at Albany, January 26-28, 1904. The president has appointed the following-named business committee: Drs. Henry A. Fairbairn, G. R. Butler, Brooklyn, and Andrew Macfarlane, Albany.

The American Röntgen Ray Society will hold its fourth annual meeting at the University of Pennsylvania, Philadelphia, December 9 and 10, 1903, under the presidency of Dr. Arthur W. Goodspeed, Philadelphia. Dr. John T. Pitkin, of Buffalo, will read a paper entitled, Dangers of the *x*-ray operator.

---

The Southern Surgical and Gynecological Association will hold its sixteenth annual meeting at the new Piedmont Hotel, Atlanta, Tuesday, Wednesday and Thursday, December 15, 16 and 17, 1903, under the presidency of Dr. J. Wesley Bovée, of Washington. Dr. Willis F. Westmoreland, of Atlanta, is chairman of the committee of arrangements. The preliminary program containing 46 papers has been issued. Reduced rates on all railroads south of the Potomac and Ohio rivers, on the certificate plan, will be granted. Any revisions of the program may be sent at once to Dr. W. D. Haggard, secretary, 302 North Vine street, Nashville, Tenn.

---

The Buffalo Academy of Medicine held meetings during the month of November, 1903, as follows:

*Section on Surgery.*—Wednesday evening, November 4. Program: Inflammatory conditions of the kidney. Also remarks on surgical operations pertaining to the kidney, George E. Fell; Contusions of lung, John Parmenter.

*Section on Medicine.*—Tuesday evening, November 10. Program: Some clinical observations on typhoid fever, Eugene Wasdin; discussion by DeLancey Rochester.

*Section on Pathology.*—Tuesday evening, November 17. Program: Protozoa as causes of disease, (yellow fever, spotted fever, smallpox, malaria and dysentery), H. G. Matzinger, of the Gratwick Laboratory; discussion led by William G. Bissell. Exhibition of specimens of pathogenic protozoa, including living trypanosomes.

---

The Buffalo Academy of Medicine will observe the following program for the remainder of the academic year (1903-4):

December 1.—Surgery. (a) Subject to be announced, A. E. Diehl; (b) The use of invisible light rays in medicine and surgery, Frederick H. Millener.

December 8.—Medicine. Stated meeting of the Academy. Subject to be announced, Irving P. Lyon.

December 15.—Pathology. Subject and place of meeting to be announced.

December 22.—Obstetrics and Gynecology. Rupture of the Uterus, Ludwig Schroeter.

December 29.—Ophthalmology, etc. Subject to be announced, Adolph H. Urban.

January 5.—Surgery. Subject to be announced, A. W. Bayliss.

January 12.—Medicine. Are we as physicians carriers of contagion? Lucien Howe.

January 19.—Pathology. Stated meeting of the Academy. Subject and place of meeting to be announced.

January 26.—Obstetrics and Gynecology. Subject to be announced, Lawrence G. Hanley.

February 2.—Surgery. Subject to be announced, Lawrence G. Hanley.

February 9.—Medicine. Tetany and its relation to gastric diseases, Allen A. Jones.

February 16.—Pathology. Subject and place of meeting to be announced.

February 23.—Obstetrics and Gynecology. Subject to be announced, C. C. Frederick.

March 1.—Surgery. Subject to be announced, E. J. Meyer.

March 8.—Medicine. Spinal cord tumors, William C. Krauss.

March 15.—Pathology. Subject and place of meeting to be announced.

March 22.—Obstetrics and Gynecology. Stated meeting of the Academy. Drainage after abdominal operations, Herman E. Hayd.

March 29.—Ophthalmology, etc. Symposium on Typhoid Fever.

April 5.—Surgery. Cutaneous tertiary syphilitic lesions, Grover W. Wende.

April 12.—Medicine. (a) Mental diseases of old age, Herman G. Matzinger; (b) Enuresis nocturna and its treatment, Julius Ullman.

April 19.—Pathology. Subject and place of meeting to be announced.

April 26.—Obstetrics and Gynecology. The relation between neurasthenia and diseases of the female generative organs, Herman B. Singer.

May 3.—Surgery. *X*-ray in diagnosis and treatment; presentation of cases; suggestions for treatment of *x*-ray burns, Sidney A. Dunham.

May 10.—Medicine. Malignant endocarditis, Henry R. Hopkins.

May 17.—Pathology. Subject and place of meeting to be announced.

May 24.—Obstetrics and Gynecology. Subject to be announced, Stephen Y. Howell.

May 31.—Ophthalmology, etc. Stated meeting of the Academy. Symposium on Tuberculosis.

June 14.—Annual meeting.

Note.—The meetings of the pathological section will be held at various laboratories in the city. It is desired that the program shall be practical in character, including exhibition of specimens and apparatus, demonstration of cultures, microscopic specimens and lantern slides. It is impossible to announce in advance the place of meeting and the subject as this will depend upon the material accessible at the time of the meeting. This information will be furnished upon the weekly postal card.

